# Polymorphic and Expressional Analysis of Genes *CDKN2B* and *ADIPOQ* in Cardiovascular Patients Using Conventional and qPCR Approach

**DOI:** 10.1155/ijhy/7379376

**Published:** 2025-08-25

**Authors:** Kashif Bashir, Sayeda Fatima Tuba Sidra Batool, Sana Zahra, Hanan Nasir, Muhammad Umar, Tooba Ashraf

**Affiliations:** ^1^Department of Biological Sciences, Superior University Lahore, Lahore 05400, Pakistan; ^2^Department of Allied Health Sciences, Superior University Lahore, Lahore 05400, Pakistan

**Keywords:** *ADIPOQ*, cardiovascular disease, *CDKN2B*, expressional analysis, polymorphisms, qPCR

## Abstract

**Background:** In the modern age, the problem of heart disease is increasing day by day which cause even more deaths than cancer. The study was designed to evaluate the polymorphism and expressional analysis of genes *CDKN2B* and *ADIPOQ* in cardiovascular patients.

**Methodology:** Blood samples of 300 cardiovascular patients and 300 controls were collected from Gannan and other hospitals of Pakistan. For polymorphism analysis, DNA was extracted followed, by conventional PCR to amplify the variants rs4977574, rs2383206, and rs2241766 of genes CDKN2B and ADIPOQ, respectively. For expressional analysis, mRNA was extracted from whole blood and converted into cDNA, followed by qPCR.

**Results:** The results show that heterozygous (AG) of rs4977574 of the *CDKN2B* gene showed highly significant association with 2-folds increased risk of cardiovascular disease (CVD) (OR = 2.03; 95% Cl = 1.26–3.25; *p* < 0.0033) while heterozygous (AG) of rs2383206 of the gene *CDKN2B* exhibited significant association but with decreased risk of CVD (OR = 0.47; 95% Cl = 0.29–0.75; *p* < 0.0017). The results of *ADIPOQ* polymorphism rs2241766 show that the heterozygous genotype (TG) showed a significant association with a decreased risk of CVD (OR = 0.63; 95% CI = 0.39–1.01; *p* < 0.05) while the homozygous mutant genotype (GG) of rs2241766 again showed a highly significant association with CVD which increased the risk of CVD by 2-folds (OR = 1.77; 95% CI = 1.11–2.80; *p* < 0.0150). The results of expressional analysis show that *CDKN2B* is significantly overexpressed in cardiovascular patients, while the gene *ADIPOQ* showed significant downregulation.

**Conclusion:** The findings show that the *CDKN2B* and *ADIPOQ* gene polymorphisms significantly raise the risk for CVD, while their expression shows a significant correlation with CVD.

## 1. Introduction

Cardiovascular disease (CVD) refers to any disease that affects the human cardiovascular system, primarily including cardiac diseases, vascular diseases of the brain and kidney, and peripheral arterial disease [1]. The leading cause of death globally is CVD. Except in Africa, it is true in all areas of the world [2]. In 2022, 17.9 million deaths (32.1%) resulted from CVD, up from 12.3 million (25.8%) in 2010 [3]. Lifestyle choices have an impact on CVD; e.g., a sedentary way of life is an important risk factor for CVD, and alcohol intake surpassing 60 g/day will increase the risk of vessel issues [4]. Physical inactivity increases the danger of many health-related problems like CVD and polygenic disorder [5]. The main factors that cause CVD are age, smoking, hypertension, high levels of lipids in blood, genetic predisposition, and other ecological factors [6]. Besides these, others include obesity, tobacco smoking, dyslipidemia, and diabetes [7]. Modern health care systems are faced with several difficulties as a result of rising industrialization, urbanization, aging of the population, and a rise in chronic diseases [8]. CVD impact 15.5 million Americans under the age of 20 [9]. The chances of getting CVD throughout one's lifetime were 49% for males and 32% for women in people aged 40, whereas the probability was 35% for men and 24% for women in people aged 70 and older [10]. Men's and women's incidence increased by more than twice as much between ages 65 and 94 and 35 to 64, respectively. CVD in parents greatly increases the disease risk by three times [11]. Genetically, this disease can occur due to either polygenic or single variant [12]. Over 40 genetic CVD can be traced to the help of a single DNA causing disease. Non-Mendelian is one of the most common CVDs and is due to innumerable genetic variants, each of which is associated with a minor effect [13]. According to genome-wide association studies (GWAS), the 9p21 region, which has been identified by the deCODE database, is a principal locus linked to CVD. An antisense long noncoding RNA mapped on the 9p21 locus is *CDKN2B*, and it has been discovered that control of cardiac *CDKN2B* expression is crucial in the development of CVD [14]. Adiponectin is located in chromosome 3q27 and which is encoded by *ADIPOQ* gene [15] and single-nucleotide polymorphisms (SNP) in *ADIPOQ* gene influences the adiponectin levels [16], adipose tissue, osteoblasts, skeletal muscle, and cardiomyocytes [17] synthesize and secrete adiponectin. This protein has antiatherogenic, cardioprotective, anti-inflammatory, and antithrombotic properties, which is one of the most abundant adipocytokines in blood [18]. Adiponectin levels were associated with the risk of CVD, which has been found by many epidemiological studies [19].

## 2. Materials and Methods

The study was organized after approval from the Superior University Ethical Committee. The study comprised 300 patients with medically confirmed CVD, together with 300 healthy individuals (controls). The blood samples of CVD patients were collected from different hospitals in Pakistan. The control blood samples were also gathered from the same hospitals. Lack of prior history of CVD was a standard for inclusion in controls, and patients with other familial diseases were also excluded. An approval form was signed by each patient before getting blood samples. Information on demographic factors was collected from each individual as well as their family history, gender, and age in detail. The phenol-chloroform method was used for DNA extraction with minor modifications done by Bashir et al. 2018 [20]. In this method, 500 μL of blood and 500 μL of solution A, which were already aliquoted, were gently combined in a 1.5 mL centrifuge tube. The combination was then kept at a temperature for roughly a quarter of an hour while the Eppendorf tube was tilted. The blood sample was then centrifuged for two minutes at 13,000 rpm. The pellet was re-added to solution A in 500 μL, followed by another two to four minutes of centrifugation at 13,000 rpm. RBC washing was carried out nearly two or three times before all of the RBCs were eliminated. Following washing, 400 μL of solution B, 25 μL of proteinase K, and 12 μL of 20% SDS solution were added to the pellet. The entire combination was allowed to incubate for 3 hours at 56°C or overnight at 37°C. Following an overnight digestion process, 500 μL of PCI was added to the mixture (phenol, chloroform, and Isoamyl alcohol). After several inversions, the Eppendorf was centrifuged for 2 min at 13,000 rpm. The bottom layer was discarded after centrifugation, and the aqueous phase was gently transferred into a different Eppendorf tube with the help of a wide-mouth pipette tip. 500 μL of solution C was combined with the aqueous layer that had been transferred to a different tube. The tube was repeatedly turned over and centrifuged at 13,000 rpm for 2–4 min. The aqueous layer was once more transferred into a different, previously labelled centrifuge tube following the centrifugation procedure. In this aqueous layer, 55 μL of sodium acetate and 500 μL of ice-chilled isopropanol solution were added. Once more, tilted and mixed for 10 min. The supernatant was carefully discarded after centrifugation to protect the particles. The pellet was centrifuged once more for three to five minutes at 13,000 rpm after being cleaned with a 500 mL solution of 70% ethanol. The pellet was carefully removed from the supernatant and allowed to air dry for two to three hours. After being thoroughly dried, the pellet was dissolved in 60 μL of TE (1 ×) solution and stored at −40°C for further use [21]. The RNA was extracted from whole blood using the Qiagen blood RNA extraction kit. The company procedure was used to extract RNA with minute modifications. The RNA quality was checked using NanoDrop. After the extraction of RNA, cDNA, and qPCR were run using CyberGreen Mastermix according to the protocol optimized by Miyata et al. [22].

For the amplification of SNPs of selected genes, 10 µL of the total reaction volume was prepared. For all polymorphism, the usage of distinct primer concentrations, MgCl_2_, concentrations, and annealing temperatures was optimized. The PCR products had been confirmed on a 2% agarose gel. Once optimized, the PCR profile used for cutting-edge experiments comprises of melting step at 94°C for 5 min, 35 cycles of 94°C for 45 s, an annealing temperature for 45 s, an extension phase at 72°C for 30 s, and a final step of extension at 70°C for 8–10 min and kept at 4°C. The specifics of the reaction mixture for natural and mutant, respectively, are shown in Table 1.

### 2.1. Statistical Analysis

Age count demographic data were provided as mean + SD. The categorical criteria of smokers/nonsmokers and yes/no, respectively, were used to assess the demographic factors of smoking status and family history. The test by Pearson was utilized to determine the genotypic and allelic frequencies of associations between patients and controls. *p* < 0.05 was calculated employing MEDCLAC. The 95% confidence intervals (CI), odds ratios (OR), and multivariate analyses were all computed by MEDCLAC.

## 3. Results

Polymerase chain reaction (PCR) was used to analyze the SNP of the *CDKN2B* and *ADIPOQ* genes in patients with CVD and healthy controls. Age, gender, smoking status, and family history were all matched between the 300 cardiac patients and the 300 healthy controls for the study.  Table 2 enlists the demographic details of the research samples and controls. The following are the main traits of study units:

Patients and health controls ranged in age from 21 to 85. Both cardiac patients and healthy controls were on average 57 years old. Age categories < 57 years and > 57 years were used to separate the cardiac patients and healthy controls. In the current study, 57% of cardiac patients were under the age of 57, and 43% were older than 57. The mean age of cardiac patients and controls showed a significant difference with up to a 2-fold increase in illness risk (OR = 2.13, 95% CI = 1.34–3.38, *p* < 0.0013). Based on gender, cardiac patients and healthy controls were split into two groups. In the case of patients, 38% of the people were female and 62% of the people were men. In the healthy control group, 52% of the individuals were female and 48% were male. According to the statistics, gender showed a significant association with CVD with a 2-fold increased risk of CVD (OR = 1.76; 95% Cl = 1.11–2.79; *p* < 0.0152. Based on their smoking habits, cardiac patients and healthy controls were divided into groups. In patients, smokers made up 76% of the population, while nonsmokers made up 24%. In the group of healthy controls, 59% were smokers and 41% were nonsmokers. Smoking showed a significant association with CVD, with a 2-fold increased risk of CVD (OR = 2.23; 95% CI = 1.35–3.66; *p* < 0.0015).

### 3.1. Polymorphism Analysis of *CDKN2B* and *ADPIOQ* Genes

For the *CDKN2B* and *ADPIOQ* gene polymorphisms, allele and genotype frequencies are given in Table 2. Genotypic distribution frequencies of the *CDKN2B* SNP rs4977574 showed that homozygous mutant (GG) showed a nonsignificant association with the CVD (OR = 0.77; 95% Cl = 0.47–1.27; *p* < 0.3170) while heterozygous (AG) of rs4977574 revealed a significant association with a 2-fold increase in the risk of CVD. (OR = 2.03; 95% Cl = 1.26–3.25; *p* < 0.003). In the case of rs2383206, the homozygous mutant (GG) showed a nonsignificant association with disease (OR = 1.32; 95% Cl = 0.84–2.08; *p* < 0.2267) while the heterozygous (AG) of rs2383206 of gene *CDKN2B* exhibited a highly significant association but with decreased risk of CVD (OR = 0.47; 95% Cl = 0.29–0.75; *p* < 0.0017). For the *ADIPOQ* gene polymorphism analysis of rs2241766, the heterozygous genotype (TG) showed a significant association with decreased risk of CVD (OR = 0.63; 95% CI = 0.39–1.01; *p* < 0.05). The homozygous mutant genotype (GG) of rs2241766 showed a highly significant association with CVD, which increased the risk of CVD by 2-fold (OR = 1.77; 95% CI = 1.11–2.80; *p* < 0.0150). Results are shown in Table 3.

Gender association with selected SNPs was also observed, which shows males with the homozygous mutant (GG) of rs4977574 showed a nonsignificant association with CVD (OR = 0.77; 95% Cl = 0.39–1.51; *p* < 0.4566). In men, the heterozygous genotype (AG) of the rs4977574 SNP of the *CDKN2B* gene showed significant association with CVD, with a higher chance of illness up to 2-fold (OR = 2.04; 95% Cl = 1.06–3.89; *p* < 0.0304). In women with the homozygous mutant (GG) genotype of rs4977574 showed nonsignificant association with CVD (OR = 0.78; 95% Cl = 0.37–1.64; *p* < 0.5141), whereas females with the heterozygous (AG) genotype showed a significant association with CVD, and the chance of illness increases up to 2-fold (OR = 2.02; 95% Cl = 0.99–4.11; *p* < 0.05). The rs2383206 SNP demonstrated a significant association in males with heterozygote (AG) by reducing the risk of CVD (OR = 0.47; 95% Cl = 0.25–0.89; *p* < 0.0219), whereas homozygous mutant (GG) of rs2383206 demonstrated a nonsignificant association with CVD. Women who had the heterozygous (AG) genotype of rs2383206 had a significantly low risk of developing CVD (OR = 0.50; 95% Cl = 0.24–1.01; *p* < 0.05). The homozygous mutant (GG) females of rs2383206 did not show any significant association with disease (OR = 1.22; 95% Cl = 0.61–2.43; *p* < 0.5591). In rs2241766 of *the ADIPOQ* gene polymorphism, the heterozygous genotype (TG) in males showed a nonsignificant association with CVD (OR = 0.66; 95% CI = 0.34–1.28; *p* < 0.2252). Homozygous mutant genotype (GG) in males showed again a nonsignificant association with CVD (OR = 1.75; 95% CI = 0.91–3.35; *p* < 0.0892). Heterozygous genotype (TG) and the mutant of rs2241766 in females showed a nonsignificant association with CVD (OR = 0.63; 95% CI = 0.31–1.26; *p* < 0.1971; OR = 1.67; 95% CI = 0.86–3.27); *p* < 0.1282). Results are shown in Table 4.

In case of age < 57, homozygous mutant (GG) and heterozygote of the rs4977574 showed nonsignificant association with CVD (OR = 0.79; 95% Cl = 0.38–1.63; *p* < 0.5330; OR = 1.93; 95% Cl = 0.96–3.88; *p* < 0.0648). In the case of age > 57, the homozygous mutant (GG) of the same SNP again showed nonsignificant association with CVD (OR = 0.80; 95% Cl = 0.40–1.62; *p* < 0.5509), while the heterozygote genotype (AG) showed a significant association with a higher risk of getting the disease up to 2-folds (OR = 1.91; 95% Cl = 0.99–3.70; *p* < 0.0530). In case of rs2383206, the heterozygote AG genotype of the *CDKN2B* gene showed significant association with a lower possibility of getting disease (OR = 0.50; 95% Cl = 0.25–1.00; *p* < 0.0520) in the age range of< 57. The homozygous mutant (GG) of the same SNP, however, showed a nonsignificantly association with CVD (OR = 1.26; 95% Cl = 0.64–2.46; *p* < 0.4990) The heterozygote AG genotype of the *CDKN2B* gene's rs2383206 reveals a significant association with a lower threat of evolving this disease in the age range of > 5 (OR = 0.48; 95% Cl = 0.25–0.94; *p* < 0.0341) while homozygous mutants (GG) of the same SNP rs2383206 indicate a nonsignificant association with the disease. (OR = 1.27; 95% Cl = 0.67–2.42; *p* < 0.4555). In rs2241766 of *ADIPOQ* gene polymorphism, the heterozygous genotype (TG) and the homozygous mutant of the age group < 54 years showed nonsignificant association with CVD (OR = 0.62; 95% CI = 0.32–1.20; *p* < 0.1612; OR = 1.84; 95% CI = 0.95–3.55; *p* < 0.0668). The heterozygous genotype (TG) and mutant of rs2241766 of age group > 54 years showed nonsignificant association with decreased risk of CVD (OR = 0.62; 95% CI = 0.30–1.28; *p* < 0.2028; OR = 1.74; 95% CI = 0.87–3.48); *p* < 0.1140). Results are shown in Table 5.

For smokers, the homozygous mutant (GG) of SNP rs4977574 showed a nonsignificant association with CVD (OR = 0.75; 95% Cl = 0.41–1.39; *p* < 0.3748), the heterozygote genotype (AG) of the rs4977574 SNP of the *CDKN2B* gene is significantly linked with CVD raising the risk up to 2-fold (OR = 2.03; 95% Cl = 1.13–3.64; *p* < 0.0171). For nonsmokers, the homozygote mutant (GG) and heterozygote of SNP rs4977574 showed nonsignificant association with CVD (OR = 0.75; 95% CI = 0.30–1.84; *p* < 0.5324; OR = 2.02; 95% CI = 0.86–4.72; *p* < 0.1031). For individuals with smoking, the heterozygote AG genotype of the rs2383206 SNP of the gene *CDKN2B* is significantly linked with reducing the chance of CVD (OR = 0.48; 95%Cl; 0.27–0.85; *p* < 0.0122) while homozygote GG mutant of this rs2383206 SNP showed a significant association with a higher chance of getting the disease by 1-fold (OR = 1.27; 95% Cl = 0.72–2.22; *p* < 0.4016). For individuals with nonsmoking, heterozygote AG genotype of rs2383206 SNP of the gene *CDKN2B* is significantly linked with reducing the chance of CVD (OR = 0.21; 95% Cl; 0.10–0.45; *p* < 0.0001), while the homozygote GG mutant of this rs2383206 SNP showed again a significant association with lessened chance (OR = 0.44; 95% Cl = 0.22–0.88; *p* < 0.0204). In rs2241766 of *ADIPOQ* gene polymorphism, the heterozygous genotype (TG) of smokers showed a nonsignificant association with decreased risk of CVD (OR = 0.61; 95% CI = 0.33–1.13; *p* < 0.1178). The homozygous mutant genotype (GG) of smokers showed a significant association with increased risk of CVD by ∼2-fold (OR = 1.82; 95% CI = 1.00–3.33); *p* < 0.0489). The heterozygous genotype (TG) and mutant of rs2241766 of nonsmokers showed a nonsignificant association with decreased risk of CVD (OR = 0.60; 95% CI = 0.27–1.30; *p* < 0.1970; OR = 1.69; 95% CI = 0.81–3.54); *p* < 0.1594). Results are shown in Table 6.

### 3.2. Expressional Analysis of *CDKN2B* and *ADPIOQ* Genes

**Figure 1:**
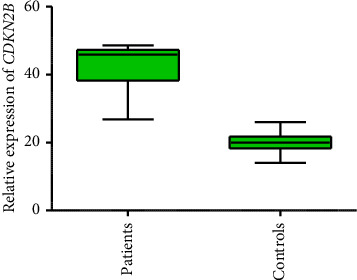
Relative expression of gene *CDKN2B* in CVD patient and controls.

**Figure 2:**
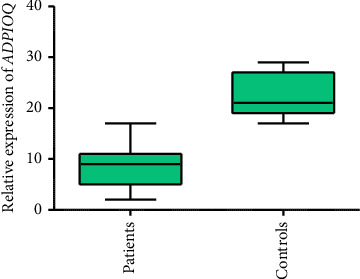
Relative expression of gene *ADPIOQ* in CVD patient and controls.

To check the expression of genes *CDKN2B and ADPIOQ* in 300 CVD patients and 300 controls, qPCR was performed. The results of qPCR showed that the expression of gene *CDKN2B* is significantly higher in patients compared to controls (*p* < 0.001). Results are shown in Figure 1, but the results of *the ADPIOQ* gene showed significant downregulation in expression in patients as compared to controls (*p* < 0.006).  Figure 2 shows the expression of gene *ADPIOQ*.

## 4. Discussion

CVD is any disease that affects the human cardiovascular system, primarily including cardiac diseases, vascular diseases of the brain and kidney, and peripheral arterial disease. CVD is the leading cause of death globally, except in Africa [22, 23]. In 2025, 18.6 million deaths (36.1%) resulted from CVD, up from 14.1 million (28.8%) in 2024 [23]. Most of the older adults are affected by CVD. In the United States, 11% of people between 20 and 40 have CVD, while 37% of people between 40 and 60, 71% of people between 60 and 80, and 85% people over 80 have CVD [24]. As far as Pakistan is concerned, some estimates of common illnesses show that the adult population consists of 41% hypertension, 17.3% with high amount of cholesterol, 10% DM and obesity, and 2.8% stroke [25].

The average age of death from coronary artery disease in the developing world is around 68, while it is around 80 in the developed world [25]. Coronary artery disease and stroke account for 75% of CVD deaths in females and 80% of CVD deaths in males [2]. Lifestyle effects have an important impact on CVD; e.g., a sedentary way of life is an important risk factor for CVD [26]. Alcohol intake exceeding 60 g/day increases the risk of cardiovascular problems [27]. Physical inactivity increases the danger of many health-related issues like CVD and diabetes [28]. Long working hours are associated with elevated CVD [29]. Long sleeping hours also affect cardiovascular health [30]. The present study was designed to observe the possible association of *CDKN2B* and *ADIPOQ* polymorphisms with CVD. In our study, the mean age of patients was 57 years. A higher number of CVD were observed in the age group < 57 years. Similar observations of the mean age trend in CVD patients have been found in Spain [31] and China [32]. Our study also indicated that males were more affected compared to females, which is supported by earlier reports from China in which the incidence of CVD was higher in males as compared to females [33]. Similar findings have also been observed in the USA [34, 35] and other countries [36]. Moreover, in this study, smoking significantly affects CVD since nonsmokers had lower CVD cases compared to smokers in this study. Similar results have been found in different studies that smoking appears to be associated with CVD [37, 38]. There is evidence that *CDKN2B* is a novel CVD susceptibility gene [39, 40]. Researchers discovered that the rs4977574 and rs2383206 polymorphisms were associated with cardiac issues and death in Taiwanese subjects without coronary lesions. It was studied that 35% of mortality in persons with normal coronary angiography was caused by CVD at a mean age of 56.6 years. However, risk increases by about 18% for each year of age, indicating that age is a predictive factor [41]. Numerous tissues, including smooth coronary muscle cells and vascular endothelial cells, have distinct expression patterns for the large antisense noncoding RNA *CDKN2B*. Noncoding RNAs during transcription and translation are part of the gene expression. There is evidence that the expression of *CDKN2B* is linked to many characteristics. Unexpectedly, the expression of *CDKN2B* is regulated by a gene variant connected to CVD. Controlling *CDKN2B* expression in the heart has been shown to alter vascular function dynamics and have a significant impact on the onset of CHD [42–44]. The polymorphisms rs4977574 and rs2383206 found in *CDKN2B* (also known as ANRIL) in chromosome 9p21.3, SNPs. This region has been considered the most extensive and found to be consistently duplicated in myocardial infarction (MI) and CVD risk loci. Although the exact function is unknown, there is a remarkable relationship between the level of *CDKN2B* mRNA and increased atherosclerosis. Changing the *CDKN2B* gene, which is vulnerable to several significant human diseases, including CVD and cancer, is mediated through expression. There is proof in studies that the new gene *CDKN2B*A raises the risk of CVD [45, 46]. Our studies indicate that polymorphisms rs4977574 and rs2383206 of the *CDKN2B*A gene showed a significant association with CVD. In the last two decades, researchers have found the involvement of rs4977574 and rs2383206 polymorphisms in developing the complex CVD. Shaner et al. found that these SNPs are involved in developing CVD and increase the risk by 2-fold [47]. Sakalar et al. also found that the SNPs rs4977574 and rs2383206 are involved in MIs in the Turkish population [48]. However, in the Turkish population, Tamel did not find any association of rs4977574 and rs2383206 polymorphism with CVD. Our results are also consistent with the Swedish population, in which rs4977574 and rs2383206 SNPs showed a significant association with increased risk of ischemic stroke and MIs [49]. However, some of the authors conducted a meta-analysis, and their results showed a strong association of rs4977574 and rs2383206 SNPs with CVD, with increased risk. But the known fact is that the polymorphic locus of these SNPs is located in the intron region of the ANRIL gene, and the question about how these SNPs affect CVD is debatable.

Adiponectin levels were associated with the risk of CVD, which has been found in epidemiology studies. Hypoadiponectinemia was associated with the prevalence of CVD, which has been demonstrated by some cross-sectional studies [50]. There is a significant inverse association between adiponectin and CVD, which has been found by prospective studies. High plasma adiponectin levels were associated with a lower risk of MI over a follow-up period of 6 years among men without previous CVD, which was found by the Health Professionals Follow-up Study. High levels of total adiponectin were associated with a lower risk of CVD among women during 14 years of follow-up, which has been recently found by the Nurses' Health Study [51]. Adiponectin is involved in CVD, in which higher levels of adiponectin protect against this disease, and low levels of adiponectin (hypoadiponectinemia) positively correlate with the risk of CVD [52–54]. Our study indicates the significant association of the *ADIPOQ* gene polymorphism rs2241766 with CVD. Zhou et al. also found the strong association of rs2241766 polymorphism with CVD. But Yang et al. did not find any association of rs2241766 with CVD. Genetic admixtures and factors from the environment in different populations tend to explain ethnicity that modulates the effect of *ADIPOQ* polymorphism on adiponectin level. The recent studies have shown that a low level of adiponectin correlates with the risk of CVD, and a high level protects against CVD [55].

## 5. Conclusions

This polymorphism and expressional study included 300 CVD patients and 300 healthy controls. The results of this study show and predict the critical expansion of CVD disease. The variants of genes *CDKN2B* and *ADIPOQ* showed a highly significant association with CVD patients. The expression of these genes shows significant upregulation and downregulation, respectively. The present study can be used as a biomarker to diagnose heart disease even before the appearance of symptoms.

## Figures and Tables

**Table 1:** PCR reaction mix composition (each reaction).

DNA template	Primer (F)	Primer (F)	PCR Mastermix	Water
1 μL (80–200 ng)	1 µL	1 µL	43 µL	4 µL

**Table 2:** Demographic table for controls and cases.

Variables	Case (*n* = 300)	Controls (*n* = 300)	Odds ratio (95% CI)	*p* value
Age (Y) mean (57) ± SD (12.9)				
< 57, *n* (%)	172 (57%)	116 (39%)	2.13 (1.34–3.38)	0.0013
> 57, *n* (%)	128 (43%)	184 (61%)		
Gender				
Male	186 (62%)	144 (48%)	1.76 (1.11–2.79)	0.0152
Female	114 (38%)	156 (52%)		
Smoking status				
Smokers	228 (76%)	176 (59%)	2.23 (1.35–3.66)	0.0015
Nonsmokers	72 (24%)	124 (41%)		

*Note: n* = total number; *p* < *x*^2^-test, chi-square test.

Abbreviations: AOR = adjusted odds ratio, CI = confidence interval.

**Table 3:** Allele and genotype frequencies of selected SNPs of gene *CDKN2B* and *ADIPOQ*.

SNPs	Geno-types	Case (*n* = 300)	Controls (*n* = 300)	Odds ratio (95% CI)	*p* value
rs4977574	AA	74 (25%)	216 (36%)	1.0 (ref)	
	AG	42 (47%)	184 (31%)	2.03 (1.26–3.25)	**0.0033**
	GG	84 (28%)	200 (33%)	0.77 (0.47–1.27)	0.3170

rs2383206	AA	54 (18%)	22 (7%)	1 (ref)	
	AG	98 (33%)	156 (51%)	0.47 (0.29–0.75)	**0.0017**
	GG	48 (49%)	126 (42%)	1.32 (0.84–2.08)	0.2267

rs2241766	TT	52 (17%)	62 (21%)	1.0 (ref)	
	TG	94 (31%)	126 (42%)	0.63 (0.39–1.01)	**0.05**
	GG	154 (51%)	112 (37%)	1.77 (1.11–2.80)	**0.01**

*Note:* OR, CI, and *p* value calculated by regression analysis. All the significant values, which are < 0.05, are shown in bold.

Abbreviations: CI, confidence interval; OR odds ratio.

**Table 4:** Association of *CDKN2B* and *ADIPOQ* polymorphisms with gender.

SNPs	Gender	Genotype	Patients *n* = 300 (%)	Controls *n* = 300 (%)	OR (95% CI)	*p* value
rs4977574	Male	Overall	186 (62%)	144 (48%)	1.76 (1.1–2.7)	0.0152
		AA	46	252	1 (ref)	
		AG	88	44	2.04 (1.06–3.8)	0.0304
		GG	52	48	0.77 (0.3–1.5)	0.4566
	Female	Overall	114 (38%)	156 (52%)	0.56 (0.35–0.8)	0.0152
		AA	28	56	1 (ref)	
		AG	54	48	2.02 (0.99–4.1)	0.0509
		GG	32	52	0.78 (0.37–1.6)	0.5145

rs2383206	Male	AA	34	10	2.99 (1.04–8.5)	0.04
		AG	60	72	0.47 (0.25–0.89)	0.0219
		GG	92	62	1.29 (0.69–2.40)	0.4136
	Female	AA	20	10	3.10 (0.99–9.65)	0.0502
		AG	38	78	0.50 (0.24–1.01)	0.0548
		GG	56	68	1.22 (0.61–2.43)	0.5591
rs2241766	Male	TT	30	28	1 (ref)	
		TG	56	54	0.66 (0.34–1.28	0.22
		GG	92	50	1.75 (0.91–3.35)	0.08
	Female	TT	22	34	1 (ref)	
		TG	38	70	0.63 (0.31–1.26)	0.19
		GG	62	64	1.67 (0.86–3.27)	0.12

*Note:* OR, CI, and *p* value calculated by regression analysis.

Abbreviations: CI, confidence interval; OR, odds ratio.

**Table 5:** Association of *CDKN2B* and *ADIPOQ* polymorphisms with age.

SNPs	Age years	Genotype	Patients *n* = 300 (%)	Controls *n* = 300 (%)	OR (95% CI)	*p* value
rs4977574	< 57	Overall	172 (57%)	116 (39%)	2.13 (1.34–3.38)	0.0013
		AA	44	42	1 (ref)	
		AG	80	36	1.93 (0.96–3.88)	0.0648
		GG	48	38	0.79 (0.38–1.63)	0.5330
	> 57	Overall	128 (43%)	184 (61%)	0.46 (0.29–0.74)	0.0013
		AA	32	66	1 (ref)	
		AG	60	58	1.91 (0.99–3.70)	0.0530
		GG	36	60	0.80 (0.40–1.62)	0.5509

rs2383206	< 57	AA	30	8	1 (ref)	
		AG	58	58	0.50 (0.25–1.00)	0.0520
		GG	84	50	1.26 (0.64–2.46)	0.4990
	> 57	AA	24	14	1 (ref)	
		AG	42	92	0.48 (0.25–0.94)	0.0341
		GG	62	78	1.27 (0.67–2.42)	0.4555

rs2241766	< 57	TT	34	26	1 (ref)	
		TG	62	52	0.62 (0.32–1.20)	0.16
		GG	100	44	1.84 (0.95–3.55)	0.06
	> 57	TT	18	36	1 (ref)	
		TG	32	74	0.62 (0.30–1.28)	0.20
		GG	54	68	1.74 (0.87–3.48)	0.11

*Note:* OR, CI, and *p* value calculated by regression analysis.

Abbreviations: CI, confidence interval; OR, odds ratio.

**Table 6:** Association of *CDKN2B* and *ADIPOQ* polymorphisms with smoking status.

SNPs	Smoking status	Genotype	Patients *n* = 300 (%)	Controls *n* = 300 (%)	OR (95% CI)	*p* value
rs4977574	Smokers	Overall	228 (76%)	176 (59%)	2.23 (1.3–3.6)	0.0015
		AA	58	64	1 (ref)	
		AG	108	54	2.03 (1.1–3.6)	0.0171
		GG	62	58	0.75 (0.4–1.3)	0.3748
	Nonsmokers	Overall	72 (24%)	124 (41%)	0.36 (0.2–0.5)	0.0001
		AA	18	44	1 (ref)	
		AG	34	38	2.02 (0.8–4.7)	0.1031
		GG	20	42	0.75 (0.3–1.8)	0.5324

rs2383206	Smokers	AA	42	12	1.0 (Ref)	
		AG	74	88	0.48 (0.27–0.85)	0.0122
		GG	116	76	1.27 (0.72–2.22)	0.4016
	Nonsmokers	AA	12	10	1.0 (Ref)	
		AG	24	62	0.21 (0.10–0.45)	0.0001
		GG	36	52	0.44 (0.22–0.88)	0.0204

rs2241766	Smokers	TT	34	32	1.0 (Ref)	
		TG	64	68	0.61 (0.33–1.13)	0.11
		GG	104	58	1.82 (1.00–3.33)	0.04
	Nonsmokers	TT	18	28	1.0 (Ref)	
		TG	30	60	0.60 (0.27–1.30)	0.19
		GG	50	54	1.69 (0.81–3.54)	0.15

*Note:* OR, CI, and *p* value calculated by regression analysis.

Abbreviations: CI, confidence interval; OR, odds ratio.

## Data Availability

Data are available on request due to privacy/ethical restrictions.
